# SLIT2/ROBO1 axis contributes to the Warburg effect in osteosarcoma through activation of SRC/ERK/c-MYC/PFKFB2 pathway

**DOI:** 10.1038/s41419-018-0419-y

**Published:** 2018-03-09

**Authors:** Shu-Jie Zhao, Yi-Fei Shen, Qing Li, Yun-Jie He, Yun-Kun Zhang, Li-Peng Hu, Yu-Qing Jiang, Nan-Wei Xu, Yu-Ji Wang, Jun Li, Ya-Hui Wang, Fei Liu, Rong Zhang, Guo-Yong Yin, Jin-Hai Tang, Dong Zhou, Zhi-Gang Zhang

**Affiliations:** 1 Department of Orthopedic, The First Affiliated Hospital of Nanjing Medical University, Nanjing, 210000 Jiangsu China; 2 Department of Orthopedics, The Affiliated Hospital of Nanjing Medical University, Changzhou No.2 People’s Hospital, Changzhou, 213003 Jiangsu China; 3 State Key Laboratory of Oncogenes and Related Genes, Shanghai Cancer Institute, Ren Ji Hospital, School of Medicine, Shanghai Jiao Tong University, Shanghai, 200240 China; 4 Department of General Surgery, The First Affiliated Hospital with Nanjing Medical University, Nanjing, 210029 Jiangsu China; 5 Department of Obstetrics and Gynecology, Shanghai Jiao Tong University Affiliated Sixth People’s Hospital, Shanghai, 200233 China; 6 Department of Obstetrics and Gynecology, Fengxian Hospital, Southern Medical University, Shanghai, 201499 China

## Abstract

Cellular metabolic reprogramming is the main characteristic of cancer cells and identification of targets using this metabolic pattern is extremely important to treat cancers, such as osteosarcoma (OS). In this study, SLIT2 and ROBO1 were upregulated in OS, and higher expression of ROBO1 was associated with worse overall survival rate. Furthermore, in vitro and in vivo experiments demonstrated that the SLIT2/ROBO1 axis promotes proliferation, inhibits apoptosis, and contributes to the Warburg effect in OS cells. Mechanistically, the SLIT2/ROBO1 axis exerted cancer-promoting effects on OS via activation of the SRC/ERK/c-MYC/PFKFB2 pathway. Taken together, the findings reveal a previously unappreciated function of SLIT2/ROBO1 signaling in OS, which is intertwined with metabolic alterations that promote cancer progression. Targeting the SLIT2/ROBO1 axis may be a potential therapeutic approach for patients with OS.

## Introduction

Osteosarcoma (OS) is the most common type of primary malignant bone tumor^1^. It typically occurs in adolescents, with a second peak of incidence in the elderly^1^. Despite advances in chemotherapy and surgery over the past 30 years, the survival rate for OS has reached a plateau^2^. A better understanding of the molecular mechanisms underlying the progression of OS is needed to advance the development of targeted therapies.

Slit guidance ligand 2 (SLIT2) binds to roundabout guidance receptor 1 (ROBO1) and plays important roles in various physiological and pathological conditions, such as axon guidance, organ development, and pro-angiogenic function^3–5^. However, conflicting reports have been published concerning the effects of this pathway in a variety of tumors^6–17^. In several studies, the SLIT2/ROBO1 axis reduced the proliferation and/or invasion of cervical, colorectal, and breast cancer cells^6–8^. Conversely, other data indicate the contribution of this pathway to tumorigenesis in nasopharyngeal carcinoma and intestinal tumors^12,15^. The biological mechanism of this axis in OS has not been reported.

A shift in glucose metabolism from oxidative phosphorylation (OXPHOS) to aerobic glycolysis is a hallmark of tumor cells^18–20^. This metabolic shift is also known as the Warburg effect and offers several advantages for tumor cell proliferation and survival, including the increased biosynthesis of macromolecules, avoidance of apoptosis, and engagement with local metabolites^21,22^. The protein family of bifunctional 6-phosphofructo-2-kinase (PFKFB) enzymes was recently identified to contribute to the Warburg effect^23,24^. Among the four isozymes (PFKFB1–4) of this family, PFKFB2 is mainly expressed in the lungs, brain, and heart^25^. Recent studies demonstrated that PFKFB2 plays a key role in several types of tumors as well as their proliferation and survival^24,26^. However, the molecular significance of the Warburg effect in the development of OS has not been fully explored. Also, it is unclear whether SLIT2/ROBO1 axis is involved in Warburg effect in OS.

In this study, we demonstrate that SLIT2 and ROBO1 are both upregulated in OS. Considering the expression of ROBO1 as determined by immunohistochemistry (IHC) staining in OS tissue sections helps to predict the overall survival rate in patients with OS. We also demonstrate that SLIT2/ROBO1 axis promotes the proliferation and inhibits the apoptosis of OS by in vitro experiments, such as cell viability, cell cycle, and cell apoptosis assays, and in vivo using a mouse xenograft model. Metabolic flux analysis revealed the contribution of the SLIT2/ROBO1 axis to the Warburg effect in OS. The data identify a novel mechanism of the SLIT2/ROBO1 axis in OS, in which SLIT2/ROBO1 regulates the SRC/ERK/c-MYC/PFKFB2 pathway to enhance the Warburg effect and facilitate the progression of OS.

## Results

### ROBO1 and SLIT2 are upregulated in OS and expression of ROBO1 is closely related to overall survival rate in OS

**Fig. 1:**
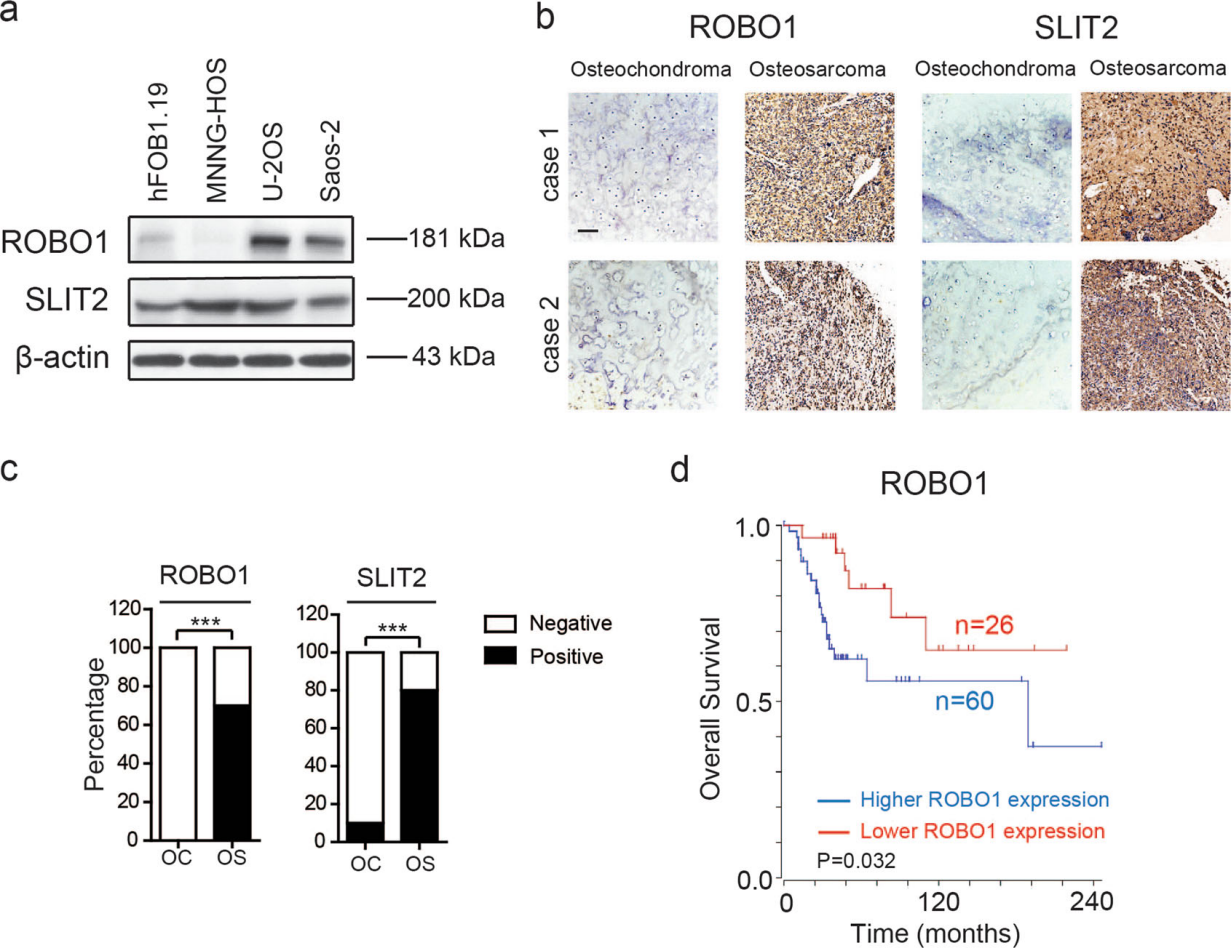
ROBO1 and SLIT2 are both upregulated in osteosarcoma (OS) and the expression of ROBO1 is closely related to the overall survival. **a** The expression patterns of ROBO1 and SLIT2 in normal osteoblast cell line (hFOB1.19) and OS cell lines (MNNG-HOS, U-2OS, and Saos-2) by western blotting. **b** The expression patterns of ROBO1 and SLIT2 in OS and osteohondroma (OC) tissues by immunohistochemical staining (IHC). Scale bars = 100 μm. **c** Statistical analysis of IHC based on the protein expression level in OS (*n* = 40) and OC (*n* = 10) tissues. **d** Kaplan–Meier analysis of overall survival rate related to the expression of ROBO1 expression in 88 OS cases based on a human osteosarcoma gene expression database (https://hgserver1.amc.nl/cgi-bin/r2/main.cgi).

To determine the expression patterns of ROBO1 and SLIT2 in OS, we first compared the expression level of ROBO1 and SLIT2 in hFOB1.19 cells (a normal human osteoblast cell line), with that in OS cell lines (MNNG-HOS, U-2OS, and Saos-2) via western blotting. Saos-2 and U-2OS cells displayed a significantly higher expression of ROBO1 (Fig. 1a), while all three OS cell lines displayed significantly higher expression of SLIT2, compared to the normal human osteoblast cell line (Fig. 1a and Supplementary Fig. 1a). Next, we detected the expression patterns of ROBO1 and SLIT2 in OS (*n* = 40) and osteochondroma (OC) (*n* = 10) tissue specimens by IHC staining. ROBO1 and SLIT2 were both expressed at significantly higher levels in OS than in OC (Fig. 1b, c). A positive correlation between the expression pattern of ROBO1 and SLIT2 was apparent (Supplementary Fig. 1c) (*n* = 40, *r* = 0.35, *p* = 0.02). To further determine the prognostic significance of ROBO1 and SLIT2 expression in patients with OS, an online human OS gene expression database was used (http://hgserver1.amc.nl). As shown in Fig. 1d and Supplementary Fig. 1d, Kaplan–Meier analysis revealed no statistically significant correlation between the expression of SLIT2 and overall survival during the follow-up period, while patients with a higher expression of ROBO1 had a significantly lower overall survival rate (*p* = 0.032). Taken together, these results indicated that ROBO1 and SLIT2 were both upregulated in OS. A high expression of ROBO1 predicted a poor overall survival rate and might facilitate the progression of OS.

### SLIT2/ROBO1 axis promotes the proliferation of OS cells and inhibits their apoptosis in vitro and in vivo

**Fig. 2:**
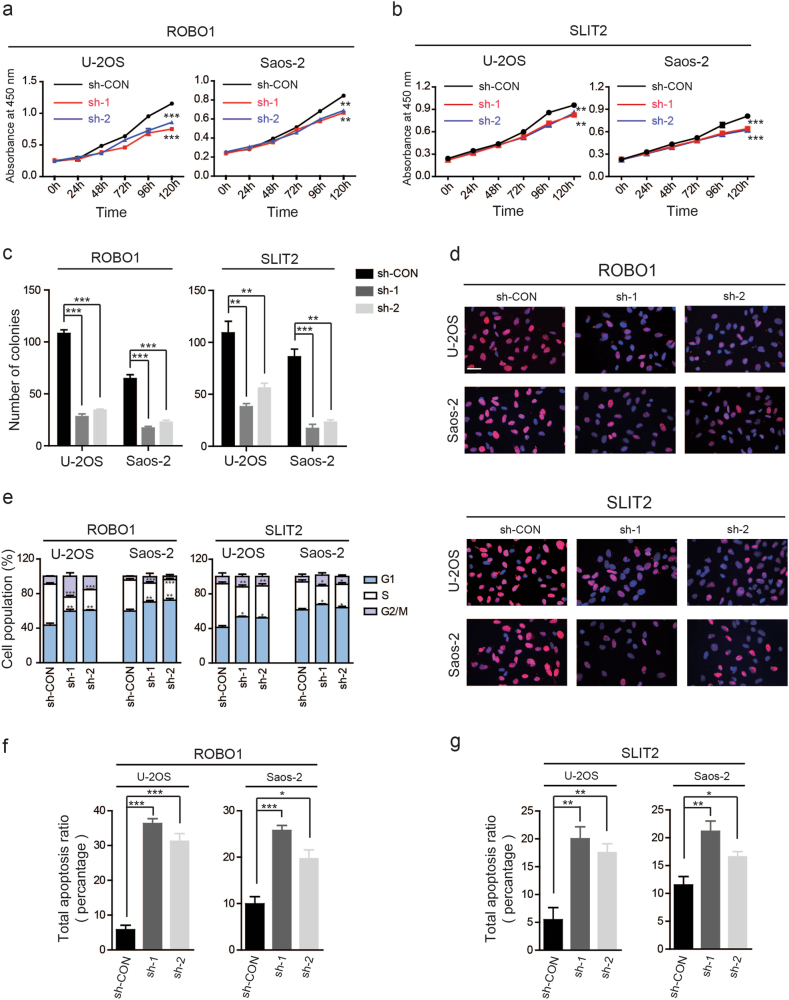
SLIT2 and ROBO1 promote proliferation and inhibits apoptosis of OS cells in vitro. **a**, **b** Knockdown of ROBO1 or SLIT2 inhibited U-2OS and Saos-2 cells proliferation using the cell counting kit (CCK)-8 assay, Values are means ± SD, **p < 0.01, ***p < 0.001. **c** Silencing of ROBO1 or SLIT2 suppressed OS cells (U-2OS and Saos-2) proliferation using colony formation assay. Values are means ± SD, **p < 0.01, ***p < 0.001. **d** Representative images of BrdU incorporation in U-2OS and Saos-2 cells transfected with ROBO1 or SLIT2 shRNA and negative control shRNA. BrdU is shown by red fluorescence, and the cell nuclei were stained with DAPI (blue fluorescence), Scale bars = 100 μm. **e** Cell cycle was determined in U-2OS and Saos-2 cells by flow cytometry after transfected with the ROBO1 sh-RNAs. The diagrams quantified cell fractions in the G_1_, S, and G_2_/M fractions were shown, *p < 0.05, **p < 0.01, ***p < 0.001. **f**, **g** Knockdown of ROBO1 or SLIT2 significantly induces U-2OS and Saos-2 cell apoptosis, *p < 0.05, **p < 0.01, ***p < 0.001.

To further test the biological functions of the SLIT2/ROBO1 axis in OS progression, we blocked this axis by targeting ROBO1 and SLIT2. sh-RNAs against ROBO1 and *SLIT2* were designed and stable knockdown cell lines (U-2OS and Saos-2 cells) were established. A non-targeting shRNA was used as control and the knockdown efficiency was confirmed by western blotting (Supplementary Figs. 2a, b). We first explored whether the knockdown of ROBO1 impaired OS growth in vitro. The results clearly indicated that the silencing of ROBO1 partly suppressed the proliferation of U-2OS and Saos-2 cells using the CCK-8 proliferation (Fig. 2a), MTT (Supplementary Fig. 2c), colony formation (Fig. 2c and Supplementary Fig. 2e), BrdU incorporation (Fig. 2d and Supplementary Fig. 2f), and cell cycle assay (Fig. 2e and Supplementary Fig. 2g). Next, we assessed the effects of ROBO1 knockdown on cell apoptosis. The stable knockdown of ROBO1 significantly induced the apoptosis of OS cells in comparison with the control cells (Fig. 2f and Supplementary Fig. 3a). We also determined the importance of SLIT2. As shown in Figs. 2b-e, g, Supplementary Figs. 2d-f, Supplementary Fig. 2h, and Supplementary Fig. 3b, the result of silencing of SLIT2 is consistent with the findings of knockdown of ROBO1 in U-2OS and Saos-2 cells.

**Fig. 3:**
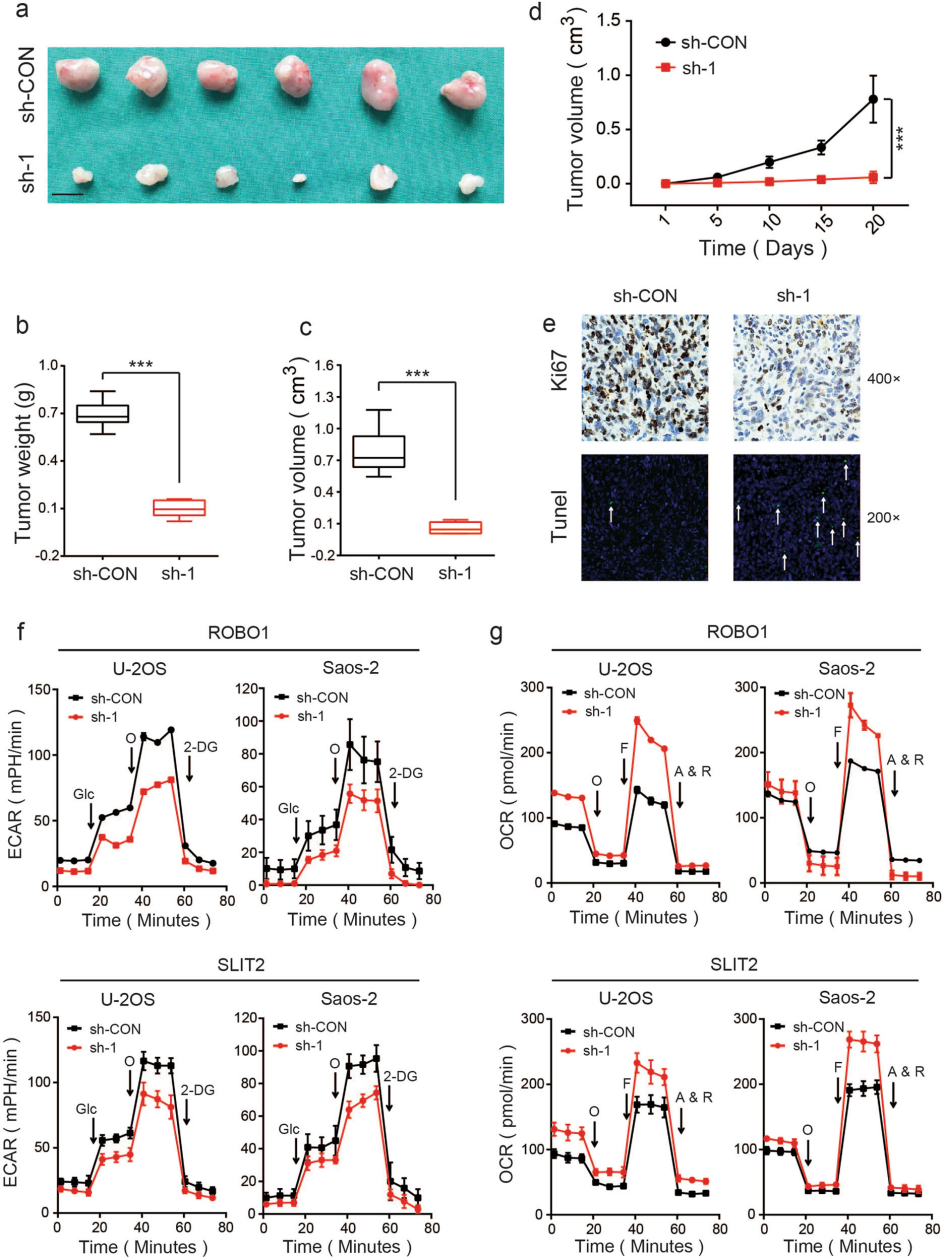
SLIT2 and ROBO1 promote OS proliferation in vivo and contribute to the Warburg effect in OS cells. **a** Morphologic characteristics of excised tumors from nude mice in U-2OS/sh-Control group and U-2OS/sh-1 group (*n* = 6). Scale bars = 1 cm. **b** Tumor weight in sh-ROBO1 group was reduced compared with sh-Control group (*n* = 6), ***p < 0.001. **c** Tumor volume in sh-ROBO1 group was reduced compared with sh-Control group (*n* = 6), ***p < 0.001. **d** Tumor volumes were measured with calipers every 5 days. The growth rate in sh-ROBO1 group was significantly slower than that in sh-Control group (*n* = 6), ***p < 0.001. **e** Representative images of Ki67 and TUNEL staining in tissues from sh-ROBO1 and sh-Control mice. A TUNEL-positive cell is indicated (arrow). Compared with sh-Control mice, decreased expression of Ki67 (upper panel) and increased TUNEL-positive cells (lower panel) were observed. **f** O_2_ consumption rate (OCR) of U-2OS or Saos-2 cells in sh-Control and sh-ROBO1 or sh-SLIT2 group was detected using a Seahorse Bioscience XFp analyzer. O Oligomycin, F FCCP, A&R antimycin A/rotenone. **g** Extracellular acidification rate (ECAR) of U-2OS or Saos-2 cells in sh-Control and sh-ROBO1 or sh-SLIT2 group was detected via a Seahorse Bioscience XFp analyzer. Glc glucose, Oligo oligomycin, 2-DG 2-deoxy-d-glucose.

Furthermore, we analyzed the proliferation-promoting and apoptosis-inhibiting properties of ROBO1 in vivo. Stable ROBO1-knockdown or control U-2OS cells were subcutaneously injected into nude mice. The stable ROBO1-knockdown group exhibited a significant delay in the growth of the xenografted tumor and a reduced tumor burden as compared with the control group (Figs. 3a–d). IHC and TUNEL assay revealed a decreased expression of the proliferation marker Ki67 and an increased rate of apoptosis in the ROBO1-knockdown xenografts (Fig. 3e). These results indicated that SLIT2/ROBO1 axis promotes the proliferation of OS cells and inhibits their apoptosis.

### SLIT2/ROBO1 axis contributes to the Warburg effect in OS cells

It is widely accepted that the Warburg effect contributes to the augmentation of survival and apoptosis avoidance in tumors^27^. A metabolic flux analyzer was used to investigate whether the SLIT2/ROBO1 axis was linked to the Warburg effect in OS. The glycolytic rate (ECAR), which is a marker of glycolysis, and mitochondrial respiration (OCR), which is a marker of oxidative phosphorylation, were measured. There was a reduction in the ECAR following the ROBO1-shRNA or SLIT2-shRNA treatment of U-2OS and Saos-2 cells (Fig. 3f and Supplementary Fig. 3c–d). In contrast, the OCR increased when ROBO1 or SLIT2 was silenced in OS cells (Fig. 3g and Supplementary Fig. 3c–d). Collectively, these data suggested that SLIT2/ROBO1 axis contributes to the induction of the Warburg effect in OS cells.

### SLIT2/ROBO1 axis regulates the expression of PFKFB2 protein via the SRC/ERK/c-MYC pathway in OS cells

**Fig. 4:**
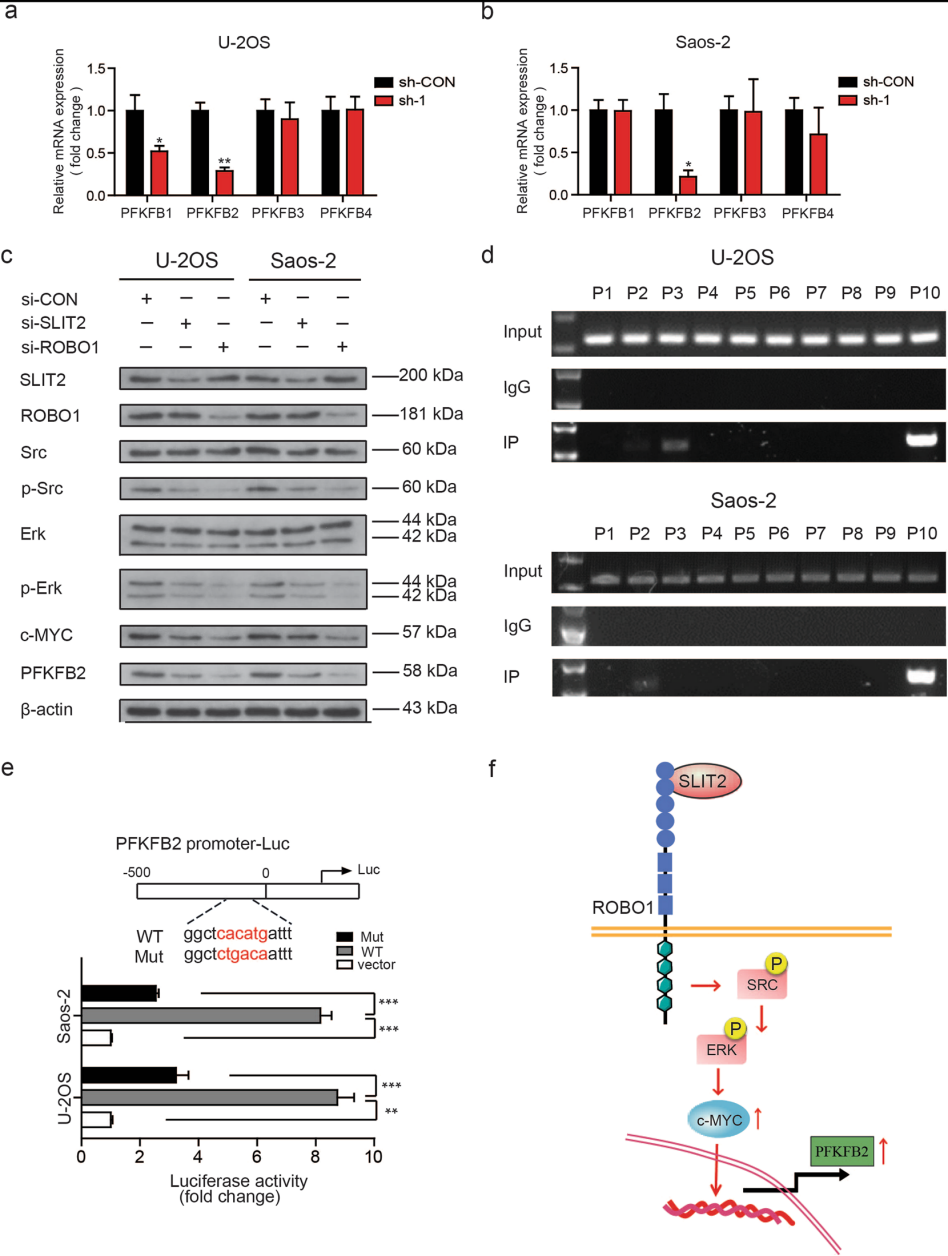
c-MYC and PFKFB2 are essential for SLIT2/ROBO1 axis-mediated Warburg effect. **a** The mRNA expression levels of PFKFBs (PFKFB1, PFKFB2, PFKFB3, and PFKFB4) in sh-Control and sh-ROBO1 U-2OS cells, Values are means ± SD, *p < 0.05, **p < 0.01. **b** The expression of PFKFBs (PFKFB1, PFKFB2, PFKFB3, and PFKFB4) was detected via real-time polymerase chain reaction (RT-qPCR) in sh-Control and sh-ROBO1 Saos-2 cells. β-actin was used as an internal control in this study. Values are means ± SD, *p < 0.05. **c** Altered protein expression level of SRC, p-SRC, ERK, p-ERK, c-MYC, and PFKFB2 was detected using western blotting upon knockdown of ROBO1 or SLIT2 in OS cells (U-2OS or Saos-2). **d** A ChIP assay was performed to confirm the potential c-MYC-binding site in the PFKFB2 promoter region in U-2OS and MNNG-HOS cell lines. IgG and input fractions were used as controls. **e** Luciferase activities of OS cells (U-2OS and Saos-2) in luciferase reporter plasmid containing wild-type and mutant PFKFB2 promoter (mutation site: red). The data shown are mean ± SD, **p < 0.01, ***p < 0.001. **f** Schematic of SLIT2/ROBO1 axis upregulated PFKFB2 expression through the SRC/ERK/c-MYC pathway in OS cells.

The bifunctional enzyme 6-phosphofructo-2-kinase (PFKFB) is involved in the synthesis and degradation of fructose-2, 6-bisphosphate (Fru-2, 6-P_2_), which is a signal metabolite that controls glycolysis^23,24^. Accordingly, targeting PFKFB enzymes appear to represent a promising approach for the treatment of certain tumors^26^. To explore the possible contributory mechanisms of ROBO1 to the Warburg effect, we first compared the gene expression patterns of PFKFB1–4 in stable *ROBO1*-knockdown and control cells using real-time polymerase chain reaction (RT-qPCR). PFKFB2 was partly downregulated in stable ROBO1-knockdown U-2OS and Saos-2 cells (Figs. 4a, b and Supplementary Fig. 4a). Consistently, a positive correlation between the expression of ROBO1 and PFKFB2 was observed among OS tissues in an online database (*r* = 0.325, *p* = 0.002) (Supplementary Fig. 4b). SRC, which is a downstream mediator of signaling through the SLIT2/ROBO1 axis, has been reported to regulate c-MYC expression via ERK activation^15,28,29^. We next asked whether SLIT2/ROBO1 signaling was related to PFKFB2 expression in OS through the SRC/ERK/c-MYC pathway. To validate this hypothesis, we confirmed that the knockdown of SLIT2 and ROBO1 by treatment with their respective specific siRNAs led to a significant reduction in the expression of phosphorylated p-SRC, p-ERK, c-MYC, and PFKFB2 in U-2OS and Saos-2 cells (Fig. 4c and Supplementary Figs. 4c–e). Furthermore, a ChIP assay was used to examine whether c-MYC could transcriptionally regulate PFKFB2 expression. As revealed in Fig. 4d, c-MYC had one common binding site in the *PFKFB2* promoter (−301 to 0 base pairs upstream of the transcription start site) in both U-2OS and Saos-2 cells. Subsequently, qPCR was used to measure the binding potential (Supplementary Fig. 4f). We then constructed luciferase reporter plasmids containing about 500 bp of the wild-type and mutant PFKFB2 promoters. The dual-luciferase reporter assays showed that the transcriptional activity of the PFKFB2 promoter was induced significantly by c-MYC in U-2OS and Saos-2 cells, and was decreased significantly in cells harboring the PFKFB2 promoter mutation construct (Fig. 4e). These results confirmed that the SLIT2/ROBO1 axis upregulated PFKFB2 expression through the SRC/ERK/c-MYC pathway in OS cells (Fig. 4f).

### PFKFB2 maintains a pro-tumorigenic phenotype and the Warburg effect in OS

**Fig. 5:**
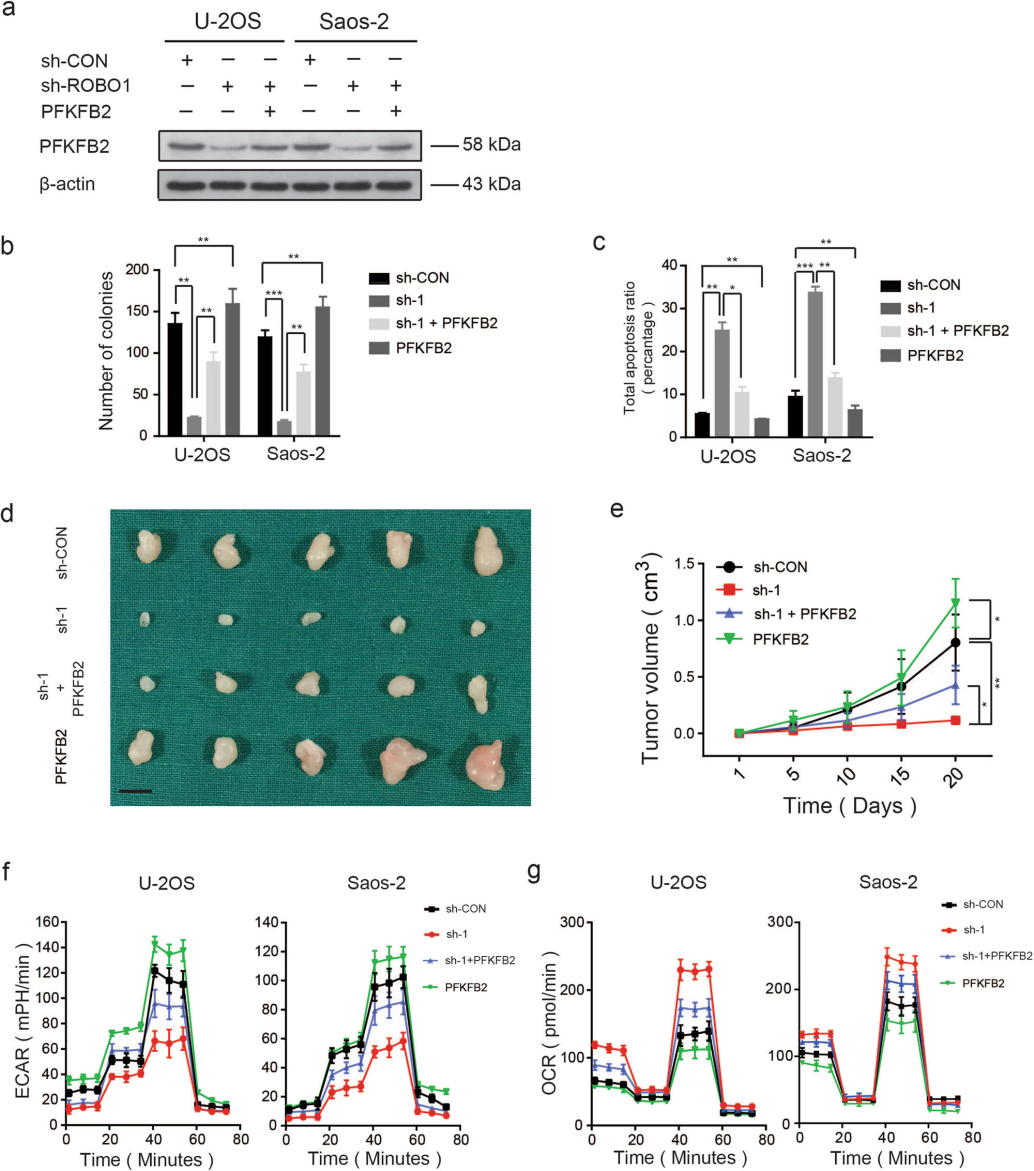
Overexpression of PFKFB2 partly rescues the effect of knockdown of ROBO1 in OS. **a** Overexpression efficacy of PFKFB2 in sh-ROBO1 OS cells (U-2OS and Saos-2) was determined by western blotting. **b** PFKFB2-overexpression partly reversed the inhibitory effects of ROBO1-knockdown on the colony formation properties of U-2OS and Saos-2 cells and overexpressed PFKFB2 in wide-type OS cells (U-2OS and Saos-2) also promoted their proliferation, values are means ± SD, **p < 0.01, ***p < 0.001. **c** PFKFB2-overexpression partly reversed the induce effect of ROBO1-knockdown on the apoptosis of OS cells (U-2OS and Saos-2). Overexpression of PFKFB2 inhibited apoptosis of wide OS cells. Values are means ± SD, *p < 0.05, **p < 0.01, ***p < 0.001. **d** PFKFB2-overexpression partly reversed the inhibitory effects of ROBO1-knockdown on the proliferation properties of U-2OS cells. Excised tumors from different groups are shown (*n* = 5). Scale bars = 1 cm. **e** PFKFB2-overexpression partly rescued the inhibitory effects of ROBO1-knockdown on the growth rate in U-2OS cells in vivo. Tumor volumes were measured with calipers every 5 days, values are means ± SD, *p < 0.05, **p < 0.01. **f** Altered level of ECAR in OS cells (U-2OS or Saos-2) in different groups (sh-Control, sh-ROBO1, sh-ROBO1 + ov-PFKFB2, and ov-PFKFB2). Values are means ± SD. **g** Altered level of OCR in OS cells (U-2OS or Saos-2) in different groups (sh-Control, sh-ROBO1, sh-ROBO1 + ov-PFKFB2, and ov-PFKFB2), Values are means ± SD.

We further evaluated whether the influence of ROBO1 knockdown on the pro-tumorigenic capacity and Warburg effect of OS could be rescued by PFKFB2 overexpression. We first overexpressed PFKFB2 in wide-type and stable ROBO1 knockdown cell lines (U-2OS and Saos-2 cells). The overexpression efficiency was then confirmed by western blotting (Fig. 5a and Supplementary Fig. 5a). As revealed by colony formation assay and cell apoptosis assay, PFKFB2 overexpression partly reversed the inhibitory effects of ROBO1 knockdown on the pro-tumorigenic properties of U-2OS and Saos-2 cells (Fig. 5b, c and Supplementary Fig. 5b, c). In an in vivo assay, a compromised tumorigenic potential in the ROBO1-knockdown group was partly offset via the overexpression of PFKFB2 (Fig. 5d, e). Similarly, the overexpression of PFKFB2 also partly maintained the Warburg effect in OS cells (Fig. 5f, g and Supplementary Fig. 5d). These results validated the functional importance of PFKFB2 as a target gene of the SLIT2/ROBO1 axis.

## Discussion

The metabolic shift termed the Warburg effect is a hallmark of tumor biology^30^. Therefore, targeting this pattern of metabolism in OS could be of therapeutic value^31,32^. In this study, we uncovered a novel mechanism through which the SLIT2/ROBO1 axis contributes to the Warburg effect by phosphorylating SRC, leading to the activation of the ERK/c-MYC/PFKFB2 pathway in OS cells.

SLIT2/ROBO1 is a conserved ligand-receptor system that is involved in cancer cell proliferation, apoptosis, migration, and angiogenesis in a cell-type dependent manner^6–17^. This axis acts as a tumor suppressor in several kinds of tumors^6–10^. However, SLIT2/ROBO1 has also been correlated with poor prognosis and has reported cancer-promoting properties^11–17^. SLIT2/ROBO1 pathway contributes to tumorigenesis and tumor growth in intestinal tumors^15^. This signaling also increases skin cancer cell proliferation by upregulating the expression of matrix metalloproteinase 2^14^. Overexpression of ROBO1 was significantly associated with worse overall and nodal relapse-free survival of nasopharyngeal carcinoma patients^12^. However, the expression profile and function of SLIT2/ROBO1 axis in OS has not been defined before.

Interestingly, the SLIT2/ROBO1 axis may have an inhibitory role during osteoblastic differentiation^33^. Inactivated osteoblast differentiation is a characteristic of OS, suggesting that the abnormal expression of SLIT2 and ROBO1 might contribute to the progression of OS. Our study confirmed that SLIT2 and ROBO1 are upregulated in OS; inhibiting SLIT2/ROBO1 signaling by the silencing of ROBO1 or SLIT2 produced an anti-tumor effect (Figs. 1a–c, 2, and 3a–e). Moreover, a Kaplan–Meier analysis revealed that patients with OS with higher expression of ROBO1 had worse overall survival rate (Fig. 1d).

The enzymes or products involved in the Warburg effect facilitate OS progression^34,35^. In this study, we tested whether the SLIT2/ROBO1 axis could promote the proliferation and apoptosis avoidance of OS cells through modulating glycolysis. PFKFB2, a member of the PFKFB family, plays a key regulatory role in glycolysis by mediating a net increase in fructose-2, 6-bisphosphate production^24^. Recent studies have also been revealed that PFKFB2 is overexpressed in a variety of tumors, although how the expression of this enzyme is mechanistically regulated during tumor progression remains unclear^36–38^. Through real-time PCR, western blot, ChIP qPCR, and luciferase assays, we identified PFKFB2 as a target gene of SLIT2/ROBO1 signaling that is directly regulated by c-MYC in OS (Figs. 4a–f).

Notably, our study reveals a new mechanism resulting from activation of the SRC/ERK/c-MYC/PFKFB2 pathway in response to crucial functions of the SLIT2/ROBO1 axis in OS. However, in ROBO1-knockdown OS cells, the overexpression of PFKFB2 partly offset the tumorigenic potential, which indicated that other molecules, such as phosphoinositol-3-kinase/Akt, E-Cadherin, and β-catenin, might be involved in the SLIT2/ROBO1 signal conduction process. More intensive studies will help to further decipher the role of SLIT2/ROBO1 axis in the development of OS. Further, in view of the low incidence of OS, more cases should be studied to conclusively analyze the clinical significance of the SLIT2/ROBO1 axis.

In conclusion, the SLIT2/ROBO1 axis facilitates the proliferation of OS cells and inhibits their apoptosis in vitro and in vivo. This cancer-promoting activity correlates with the Warburg effect through the activation of the SRC/ERK/c-MYC/PFKFB2 pathway. Our findings provide a novel insight into the biology of OS progression and raise the possibility that targeting the SLIT2/ROBO1 axis could be a beneficial clinical anti-tumor strategy for OS patients.

## Materials and methods

### Cell culture and reagents

Human osteoblast hFOB1.19 cells and human osteosarcoma Saos-2 and MNNG-HOS cells were obtained from the Cell Bank of the Chinese Academy of Sciences (Shanghai, China). Human osteosarcoma U-2OS cell line was obtained from the American Type Culture Collection (ATCC, Manassas, VA, USA). Each cell line passed the test of DNA profiling (STR). Mycoplasma contamination testing was performed using Mycoplasma Genus PCR. All cell lines were cultured according to ATCC instructions. Standard culture conditions were used for the human osteosarcoma cells (37 °C in a 5% CO_2_ atmosphere), while the hFOB1.19 cells were incubated at 34.5 °C in a 5% CO_2_ atmosphere. Cells were passaged 7–10 times. The antibodies used were ROBO1 (ab7279; Abcam, Cambridge, UK), SLIT2 (ab134166; Abcam), SRC (2109; Cell Signaling Technology, Danvers, MA, USA), p-Src (12432; Cell Signaling Technology), extracellular signal-regulated kinase (ERK, 4695; Cell Signaling Technology), p-ERK (4370; Cell Signaling Technology), c-Myc (ab32072; Abcam), PFKFB2 (17838; Proteintech, Wuhan, China), β-actin (M1210-2; Hua’an Biology, Chuzhou, China), BrdU (66241-1-1g, Proteintech, Wuhan, China), horseradish peroxidase-conjugated anti-rabbit IgG (170-6515; Bio-Rad Laboratories, Hercules, CA, USA), and horseradish peroxidase-conjugated anti-mouse IgG (170-6516, Bio-Rad Laboratories).

### Database analysis

We performed Kaplan–Meier analysis of 88 OS patients using an online database (http://hgserver1.amc.nl). The data from this database were used to perform a correlation analysis between the gene expression levels of ROBO1 and PFKFB2.

### siRNA transfection

U-2OS and Saos-2 cells were cultured at 60% confluence and transfected with ROBO1-specific small interfering (si)RNA, SLIT2-specific siRNA, or a negative control siRNA (GenePharma, Shanghai, China) using Lipofectamine^®^ RNAiMAX (Thermo Fisher Scientific, Waltham, MA, USA). The transfection procedures were performed according to the manufacturer’s protocols. The sequences of these siRNAs are listed in Supplementary Table 1.

### Plasmid transfection

The sequences of the short hairpin (sh)RNAs targeting ROBO1 were sh-1, 5′-GCACTGGACAGTAGATCAACA-3′ and sh-2, 5′-GGATCATCCTCATGGTCTTCA-3′. The short hairpin (sh)-SLIT2 sequences were sh-1, 5′-CCGGCCTCACCTTAATTCTTAGTTACTCGAGTAACTAAGAATTAAGGTGA GGTTTTTG-3′ and sh-2, 5′-CCGGCCTGGA GCTTTCTCACCATATCTCGAGATATGGTGAGAAAG CTCCAGGTTTTTG-3′. The shRNA-containing plasmids and a negative control plasmid were purchased from GenePharma (Shanghai,China). The plasmid containing PFKFB2-HA and a negative control plasmid were obtained from FulenGen Ltd., Co. (Guangzhou, China). All these plasmids were packaged into virus particles using HEK 293T cells and the viral titers were determined. To establish stable ROBO1-knockdown cell lines or PFKFB2-overexpressing cells, the target cells were infected with 1 × 10^8^ lentivirus-transducing units with 6 μg/mL polybrene (Sigma-Aldrich, St. Louis, MO, USA). The infected cells were then screened with 2.5 μg/mL puromycin after 72 h. The efficiency of the knockdown or overexpression was verified by western blotting.

### Quantitative real-time PCR (qPCR)

Total RNA extraction and reverse transcription were performed as described before^39^. The primer sequences are listed in Supplementary Table 1. Quantification of all gene transcripts was performed with the SYBR^®^ Premix Ex Taq™ kit (Takara Bio, Ōtsu, Japan). The settings of the thermal cycler program have been previously described^39^. β-actin and 18S were used as internal controls.

### Western blotting

Western blotting was performed as previously described^39^. In brief, whole cellular proteins were extracted and equal amounts of proteins were subjected to 10% sodium dodecyl sulfate-polyacrylamide gel electrophoresis. After electro-transfer of the proteins and blocking, the membranes were incubated with the indicated primary antibodies overnight at 4 °C. After incubation with the secondary antibodies, binding was detected using western ECL substrate (Share-bio, Shanghai, China).

### IHC staining

A microarray containing tissue from 40 OS patients was obtained from Alena Biotechnology Co., Ltd. (Xi’an, China). The IHC assay was performed as previously described^39^. Specially, we performed immunohistochemistry with only the first antibody or only the secondary antibody as controls (Supplementary Fig. 1b). ROBO1 and SLIT2 were detected using the corresponding primary antibodies at 1:100 dilutions. All the sections were photographed using a fluorescence microscope (Carl Zeiss, Oberkochen, Germany). The intensities of ROBO1 and SLIT2 staining were scored using the following criteria: 0–5% staining was scored as 0; 6–35% staining was scored as 1; 36–70% staining was scored as 2; and >70% staining was scored as 3. A total score <2 was considered to represent negative expression, and a score ≥2 was defined as positive expression. The scoring was performed in a blinded manner and determined by two senior pathologists.

### CCK-8, MTT, and colony formation assays

We performed the CCK-8 assay following the vendor’s instructions (Dojindo Molecular Technologies, Japan). Briefly, target cells (3 × 10^3^) were seeded in 96-well plates. The optical absorbance at 450 nm was detected using a plate reader (Thermo Fisher Scientific) at 0, 24, 48, 72, 96, and 120 h.

For the MTT assay, cells were first seeded into 96-well plates at a density of 4 × 10^3^ cells in 200 μL of complete culture medium. After 24, 48, 72, and 96 h incubation, 25 μL of a 3-(4,5-dimethylthiazol-2-yl)-2,5-diphenyltetrazolium bromide (MTT, Sigma-Aldrich, St. Louis, MO, USA) solution at a concentration of 5 mg/mL and pH 7.4 was added to each well and the cells were incubated for 4 h. Then, 150 μL dimethyl sulfoxide was added to each well and the plate was lightly shaken for 10 min at room temperature. The absorption at 570 nm was measured using a plate reader (Thermo Fisher Scientific).

For the colony formation assay, the target cells (1 × 10^3^) were seeded per well of a 6-well plate. After 2 weeks, colonies were fixed with paraformaldehyde and stained with 0.5% (w/v) crystal violet. Photographs were acquired and the cell numbers were counted.

### BrdU cell proliferation assay

Cells were cultured in 12-well chambers (Ibidi GmbH, Planegg, Germany) for cell immunofluorescence staining. The cells were treated with BrdU (Yeasen, Shanghai, China) at a concentration of 10 μM for 48 h. Thereafter, the cells were fixed with 4% polyformaldehyde for half an hour at room temperature. After a wash in 2× PBS, the cells were then incubated for 5 min at 37 °C in 2 mol/L HCl. Thereafter, the cells were equilibrated at room temperature for 10 min in borate buffer, permeabilized with 0.1% Triton X-100 for 2 min, and blocked with 3% bovine serum albumin (BSA) for 60 min at room temperature. The cells were incubated overnight with primary antibody against BrdU at 4 °C and then labeled with Alexa 594-conjugated secondary antibody (1:200 dilution) for 1 h at room temperature. The nuclei were counterstained for 2 min with 4′,6-diamidino-2-phenylindole (DAPI; Sigma-Aldrich). Images were acquired using confocal microscopy with a LSM 510 METALaserScanning Microscope (Carl Zeiss, Jena, Germany).

### Cell apoptosis assay

A cell apoptosis assay was performed using an Annexin V/propidium iodide apoptosis kit (BD Biosciences, Franklin Lakes, NJ, USA) following the manufacturer’s protocol. Adherent cells were cultured in serum-free medium for 24 h. The cells were detached with 0.25% trypsin (without ethylenediaminetetraacetic acid), washed, re-suspended with binding buffer, and stained with annexin V-fluorescein isothiocyanate and propidium iodide. The percentages of Annexin V-positive and propidium iodide-negative cells were determined by flow cytometry using a FACS Cater -plus flow cytometer (BD Biosciences).

### Cell cycle analysis

Cells were seeded into six-well plates, cultured for 24 h, washed with cold PBS, and fixed in pre-cold 70% ethanol at 4 °C overnight. The cells were recovered by centrifugation at 1000 rpm for 5 min and washed with PBS for 15 min. Thereafter, 1 mL DNA staining solution and 10 µL permeabilization solution (MultiSciences Biotech Co, HangZhou, China) was added and the cells were incubated in the dark at room temperature for 30 min. Cell cycle was determined and analyzed by flow cytometry using the aforementioned flow cytometer.

### Mouse xenograft model

The BALB/C nude mice (male, 5-weeks-old) were reared and all the animal experiments were done following the animal experimental protocols approved by the East China Normal University Animal Care Commission. The mice were first randomly divided into several groups and subcutaneously injected with 1.5 × 10^6^ of the target cells. The size of the tumor was recorded every 5 days. After 20 days, all mice were killed and the tumors were isolated. The volume and weight of each harvested xenografted tumor were measured. Then, the tissue samples were fixed and prepared for further histological analysis.

### Terminal deoxynucleotidyl transferase (TdT) dUTP nick-end labeling (TUNEL) assay

A TUNEL kit (Roche, Basel, Switzerland) was used to quantify the proportion of apoptotic cells in tissue sections from the xenograft tumors. We performed this assay following the protocols provided by the manufacturer. In brief, the tissue sections were deparaffinized and rehydrated, then blocked to inactivate endogenous peroxidases. Next, the slides were permeabilized and incubated in a reaction mixture including terminal deoxynucleotide transferase and deoxyuridine triphosphate for 2 h at 37 °C. The nuclei were counterstained with DAPI (Sigma-Aldrich) and images were acquired using a fluorescent microscope (Carl Zeiss).

### Measurement of oxidative phosphorylation and glycolysis

The metabolic alternations of cultured cells were monitored using an XF96 metabolic flux analyzer (Seahorse Biosciences, Billerica, MA, USA) according to the manufacturer’s instructions. The ROBO1 or SLIT2 siRNA-transfected and negative control siRNA-transfected cells were plated in wells of a model XF96 96-well plate (Seahorse Biosciences; 3 × 10^4^ cells in the 80 μL added to each well) and incubated overnight at 37 °C. The XF calibration solution (Seahorse Biosciences) was added to the XF sensor cartridge (Seahorse Biosciences) and was also incubated at 37 °C overnight but without CO_2_. The next day, prior to the assay the complete medium was replaced with XF assay modified DMEM medium (1 g/mL glucose, pH 7.4; Seahorse Biosciences) and the cells were incubated at 37 °C for 1 h without CO_2_. The real-time glycolytic rate (ECAR) was assessed by the sequential injection of 10 mM glucose, 1 mM oligomycin (Sigma-Aldrich), and 80 mM 2-deoxyglucose (D8375; Sigma-Aldrich). The OCR was assessed by the sequential injection of 1 mM oligomycin, carbonyl cyanide 4-(trifluoromethoxy) phenylhydrazone (C2920; Sigma-Aldrich, C2920), 2 mM antimycin A, and 2 mM rotenone (Sigma-Aldrich). The instrument recorded 12 measurements for ECAR (nmoles/min) and OCR (mpH/min) in each well. The results were analyzed using the XFe Wave software (Seahorse Biosciences).

### ChIP assay

U-2OS and Saos-2 cells were fixed with 1% (w/v) formaldehyde solution for 10 min at 37 °C. Subsequent steps were performed using the Pierce™ agarose ChIP kit (Thermo Fisher Scientific) following the manufacturer’s instructions. A c-MYC-specific antibody or rabbit IgG isotype were incubated with the fixed cells overnight at 4 °C. PCR was performed with the input DNA or the immunoprecipitates using SYBR^®^ Green master mix and primers (Takara, Japan). The primers used for the ChIP assay are listed in Supplementary Table 2. Additionally, qPCR was performed to detect the binding potential; the specific primers are listed in Supplementary Table 2.

### Luciferase reporter assay

To verify the binding sites of c-MYC and PFKFB2, luciferase reporter plasmids containing wild-type and mutant PFKFB2 promoters were cloned in the PGL4.10 vector (Supplementary Table 2). U-2OS or MNNG-HOS cells were transiently co-transfected with c-MYC and PGL4.10-PFKFB2 vectors using Lipofectamine 3000 (Invitrogen, Carlsbad, CA, USA). Cells were collected 48 h after transfection and luciferase activity was analyzed with the Dual-Luciferase Reporter Assay System (Promega, Madison, WI, USA). *Renilla* luciferase activity was used to normalize for transfection efficiency. The data are presented as the fold-change relative to the control group.

### Statistical analyses

Data are presented as mean ± standard deviation (SD). Statistical analyses were done using Prism 5.0 for Windows (GraphPad Software, La Jolla, CA, USA). The chi-square test (SPSS 17.0; IBM, Armonk, NY, USA) was used to evaluate the correlation between the expression of ROBO1 and SLIT2 in patients with OS. Spearman’s rank correlation test was used to analyze the relationship between the expression of ROBO1 and PFKFB2. Comparisons between groups were performed by two-tailed Student’s *t*-test. Values of *p* < 0.05 were considered statistically significant.

## Electronic supplementary material

Supplementary Figlegends

Supplementary figure 1

Supplementary figure 2

Supplementary figure 3

Supplementary figure 4

Supplementary figure 5

Supplementary Table 1

Supplementary Table 2
