# Supplementary material for: SLIT2/ROBO1 axis contributes to the Warburg effect in osteosarcoma through activation of SRC/ERK/c-MYC/PFKFB2 pathway
Source: Cell Death Dis. 2018 Mar 9;9(3):390. doi: 10.1038/s41419-018-0419-y (PMC5844886; doi:10.1038/s41419-018-0419-y)
Supplement: Supplementary file 1 — Supplementary Figlegends [file 41419_2018_419_MOESM1_ESM.doc]

**Supplementary Fig 1**

1. The quantification of SLIT2 bands in hFOB1.19 cells, with that in OS cell lines

(MNNG-HOS, U-2OS, and Saos-2 cells).

(b) Immunohistochemical staining (IHC) of OS tissues with only the first antibody or with only the secondary antibody. Scale bars = 200 μm.

(c) A positive correlation between the expression pattern of ROBO1 and SLIT2 (*n* = 40, *r* = 0.35, *p* = 0.02). Representative photographs of the expression patterns of ROBO1 and SLIT2 in OS were shown in the left panel. Scale bars = 100 μm.

(d) Kaplan-Meier analysis of overall survival rate related to the expression of SLIT2 expression in 88 OS cases based on a human osteosarcoma gene expression database (https://hgserver1.amc.nl/cgi-bin/r2/main.cgi).

**Supplementary Fig 2**

(a and b) Interference efficacy of sh-RNA targeting of ROBO1 and SLIT2 in U-2OS and Saos-2 cells was detected by western blotting.

(c and d) Knockdown of ROBO1 or SLIT2 inhibited U-2OS and Saos-2 cells proliferation using the cell counting MTT assay, Values are means ± SD, ***p* < 0.01, ****p* < 0.001.

(e) Representative photographs of the colony formation assay in U-2OS and Saos-2 cells transfected with ROBO1 or SLIT2 shRNA and negative control shRNA.

(f) SLIT2 or ROBO1 shRNA decreased the percentage of BrdU-positive OS cells (U-2OS and Saos-2). Values are means ± SD, ***p* < 0.01, ****p* < 0.001.

(g) Representative photographs of the cell cycle assay in U-2OS and Saos-2 cells transfected with ROBO1 shRNA or negative control shRNA.

(h) Representative photographs of the cell cycle assay in U-2OS and Saos-2 cells transfected with SLIT2 shRNA or negative control shRNA.

**Suplementary Fig 3**

(a) Representative images of OS cellular apoptosis ability in sh-Control and sh-ROBO1.

(b) Representative images of OS cellular apoptosis ability in sh-Control and sh-SLIT2.

(c) The ratio of basal ECAR/OCR of OS cells transfected with sh-ROBO1, sh-SLIT2 and sh-Control were measured. Values are means ± SD, **p* < 0.05, ***p* < 0.01, ****p* < 0.001.

(d) The ratio of maximal ECAR/OCR of OS cells transfected with sh-ROBO1, sh-SLIT2 and sh-Control were measured. Values are means ± SD, **p* <0.05, ***p* < 0.01, ****p* < 0.001.

**Suplementary Fig 4**

(a) The expression of PFKFBs (PFKFB1, PFKFB2, PFKFB3 and PFKFB4) were detected via real-time polymerase chain reaction (PCR) in sh-Control and sh-ROBO1 Saos-2 cells, 18S were used as an internal control in this study. Values are means ± SD, **p* < 0.05, ***p* < 0.01.

(b) A correlation analysis between ROBO1 and PFKFB2 expression level by analyzing OS gene expression database (https://hgserver1.amc.nl/cgi-bin/r2/main.cgi).

(c) Interference efficacy of si-RNA targeting of ROBO1 and SLIT2 in OS cells (U-2OS and Saos-2) was detected by real-time qPCR (*n* = 3). We used 18S as an internal control in this study. Values are means ± SD, ****p* < 0.001.

(d) Interference efficacy of si-RNA targeting of ROBO1 and SLIT2 in OS cells (U-2OS and Saos-2) was detected by real-time qPCR (*n* = 3). β-actin was regarded as an internal control in this assay. Values are means ± SD, ***p* < 0.01, ****p* < 0.001.

(c) The quantification of western bands in Fig. 4c.

(f) ChIP-qPCR detection of c-MYC binding regions (ChIP-Seq peaks) in the U-2OS and Saos-2 cells. Values are means ± SD, ****p* < 0.001.

**Supplementary Fig 5**

(a) Overexpression efficacy of PFKFB2 in wild OS cells (U-2OS and Saos-2) was determined by western blotting.

(b) Representative images of OS cellular apoptosis ability in sh-Control, sh-ROBO1, sh-ROBO1 + ov-PFKFB2 and ov-PFKFB2 groups are shown.

(c) Representative images of OS colony formation assay in sh-Control, sh-ROBO1, sh-ROBO1 + ov-PFKFB2 and ov-PFKFB2 groups are shown.

(d) The ratio of basal ECAR/OCR and maximal ECAR/OCR in sh-Control, sh-ROBO1, sh-ROBO1 + ov-PFKFB2 and ov-PFKFB2 groups were measured. Values are means ± SD, **p* < 0.05, ***p* < 0.01, ****p* < 0.001.
