# Supplementary figures and images for: SLIT2/ROBO1 axis contributes to the Warburg effect in osteosarcoma through activation of SRC/ERK/c-MYC/PFKFB2 pathway
Source: Cell Death Dis. 2018 Mar 9;9(3):390. doi: 10.1038/s41419-018-0419-y (PMC5844886; doi:10.1038/s41419-018-0419-y)

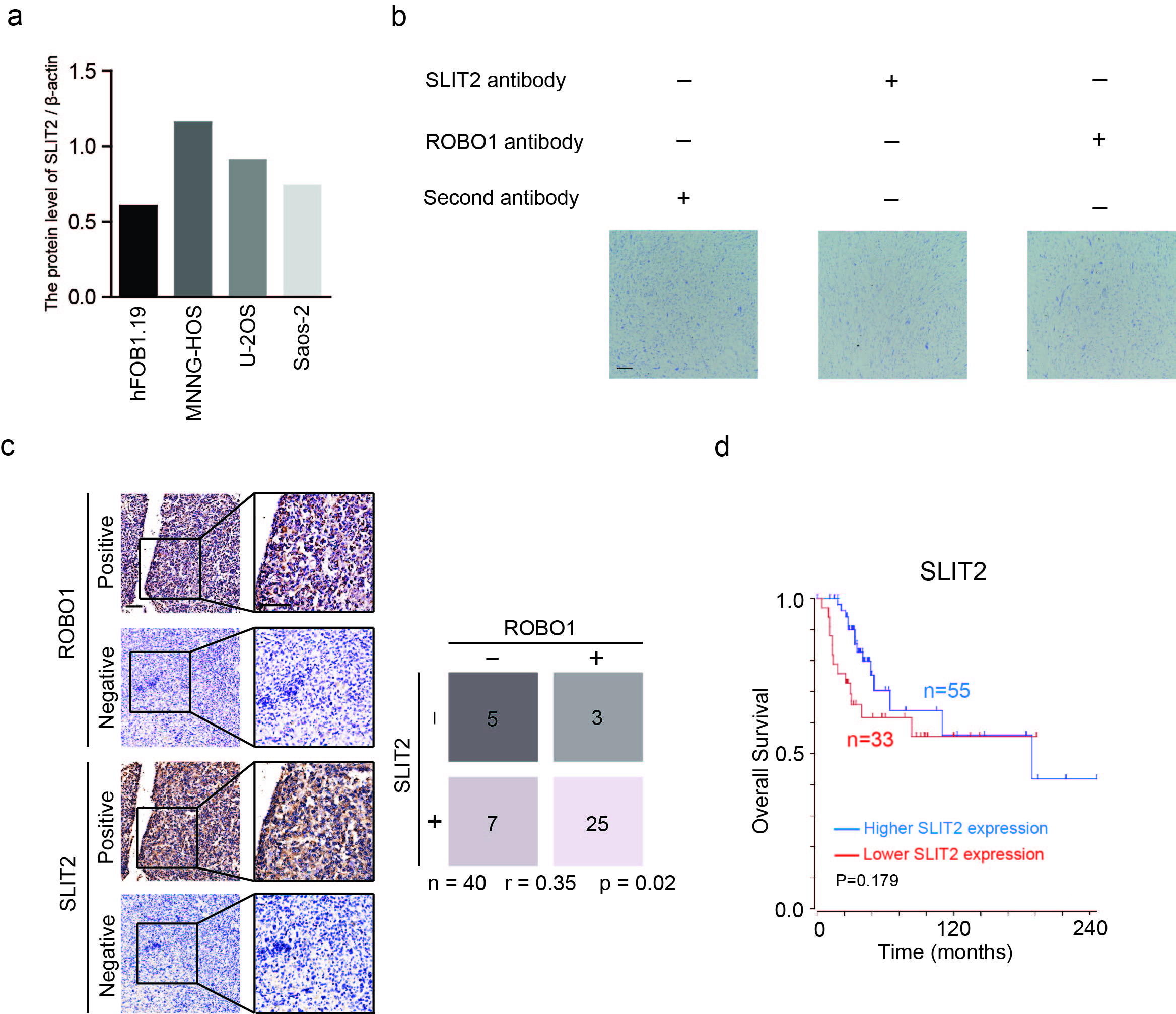

Supplement: Supplementary file 2 — Supplementary figure 1 [file 41419_2018_419_MOESM2_ESM.jpg]

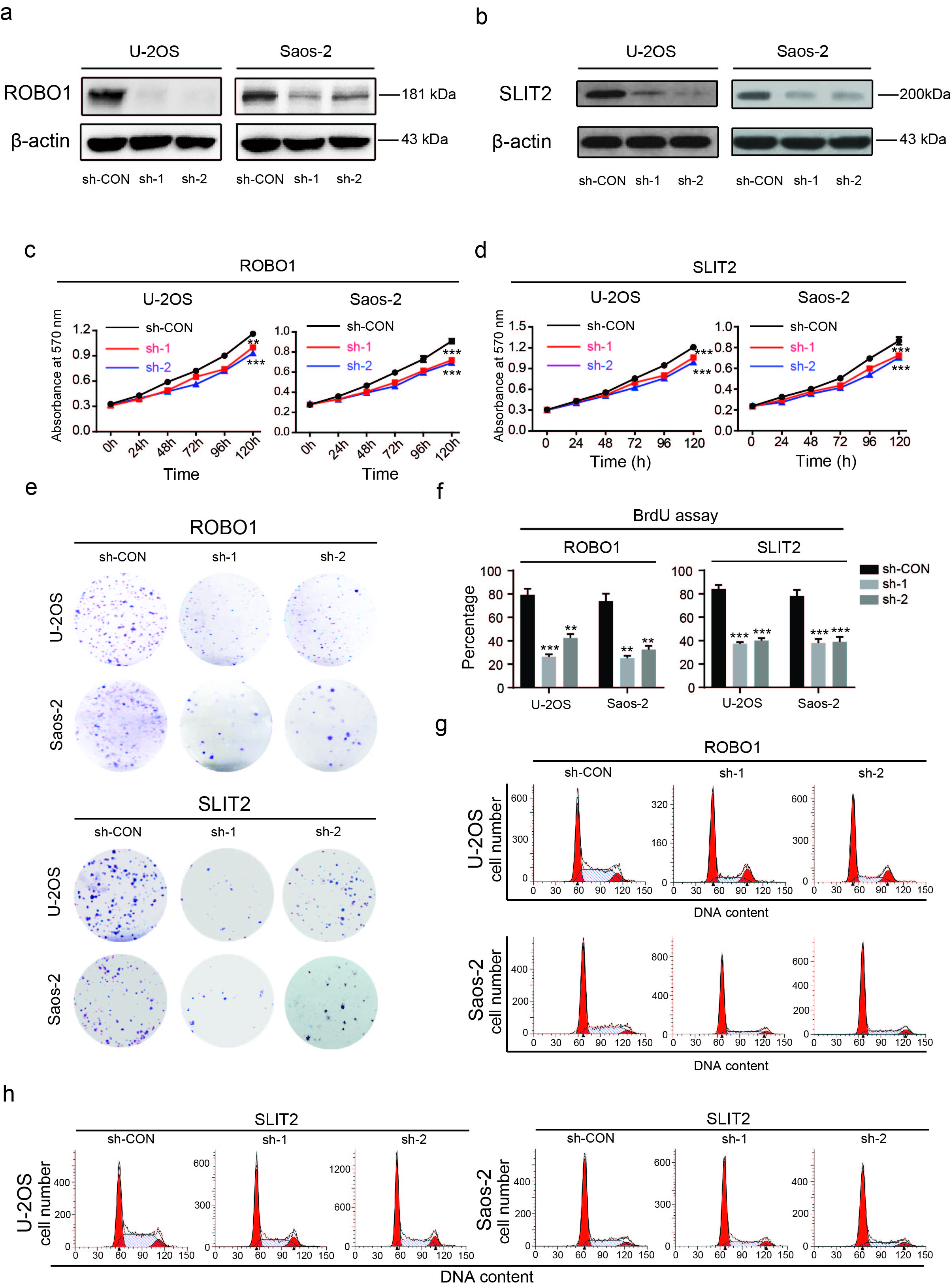

Supplement: Supplementary file 3 — Supplementary figure 2 [file 41419_2018_419_MOESM3_ESM.jpg]

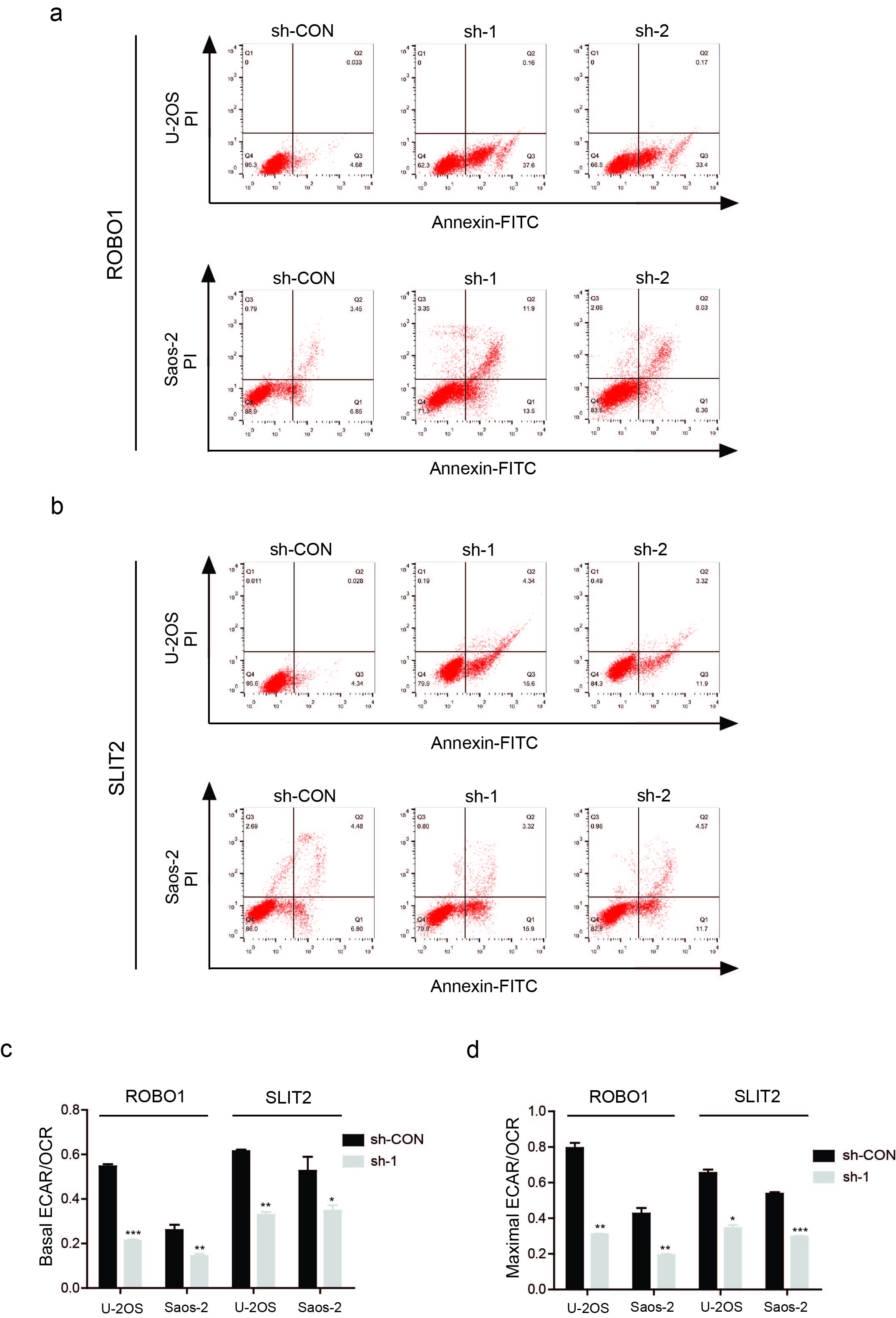

Supplement: Supplementary file 4 — Supplementary figure 3 [file 41419_2018_419_MOESM4_ESM.jpg]

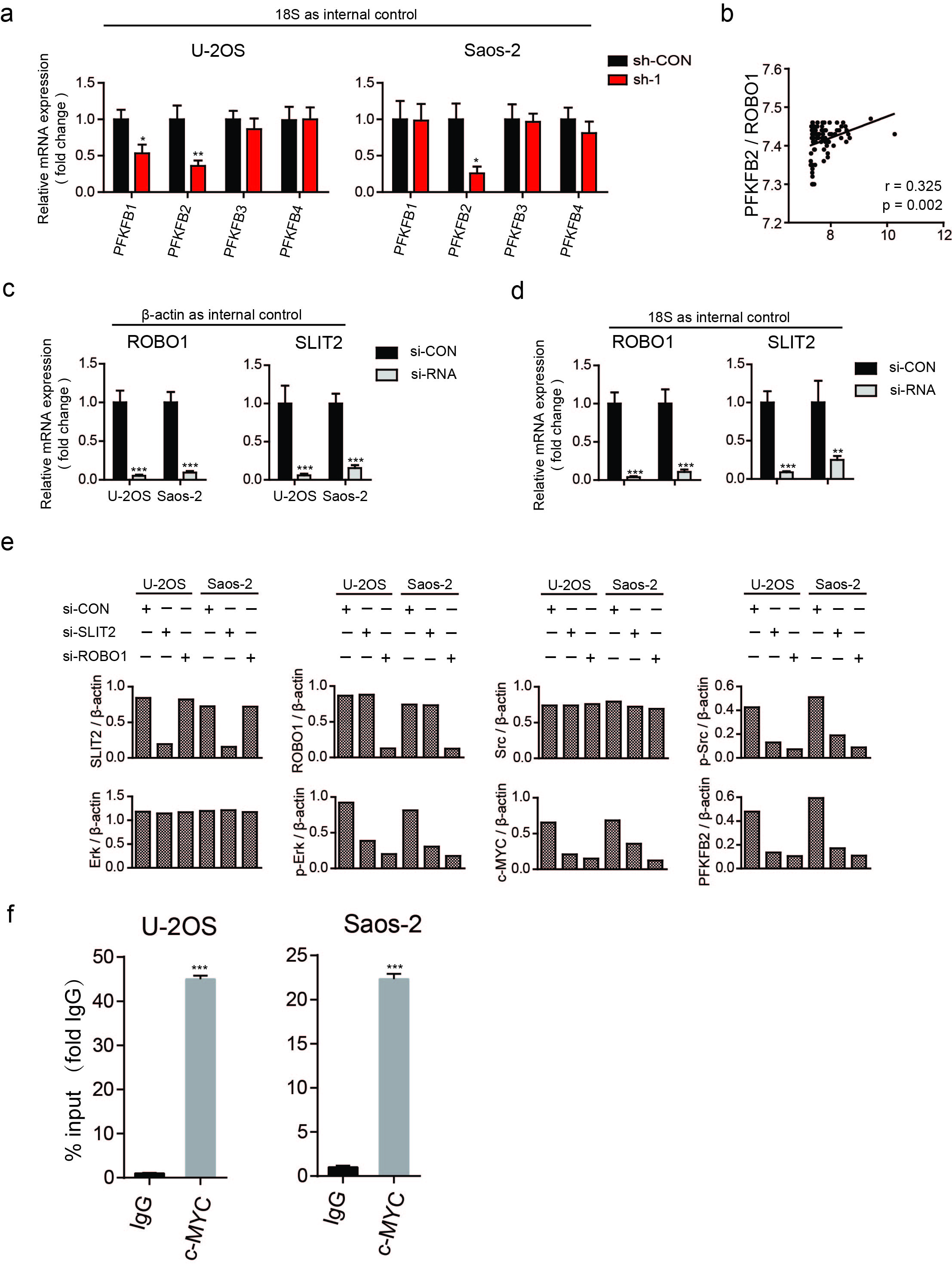

Supplement: Supplementary file 5 — Supplementary figure 4 [file 41419_2018_419_MOESM5_ESM.jpg]

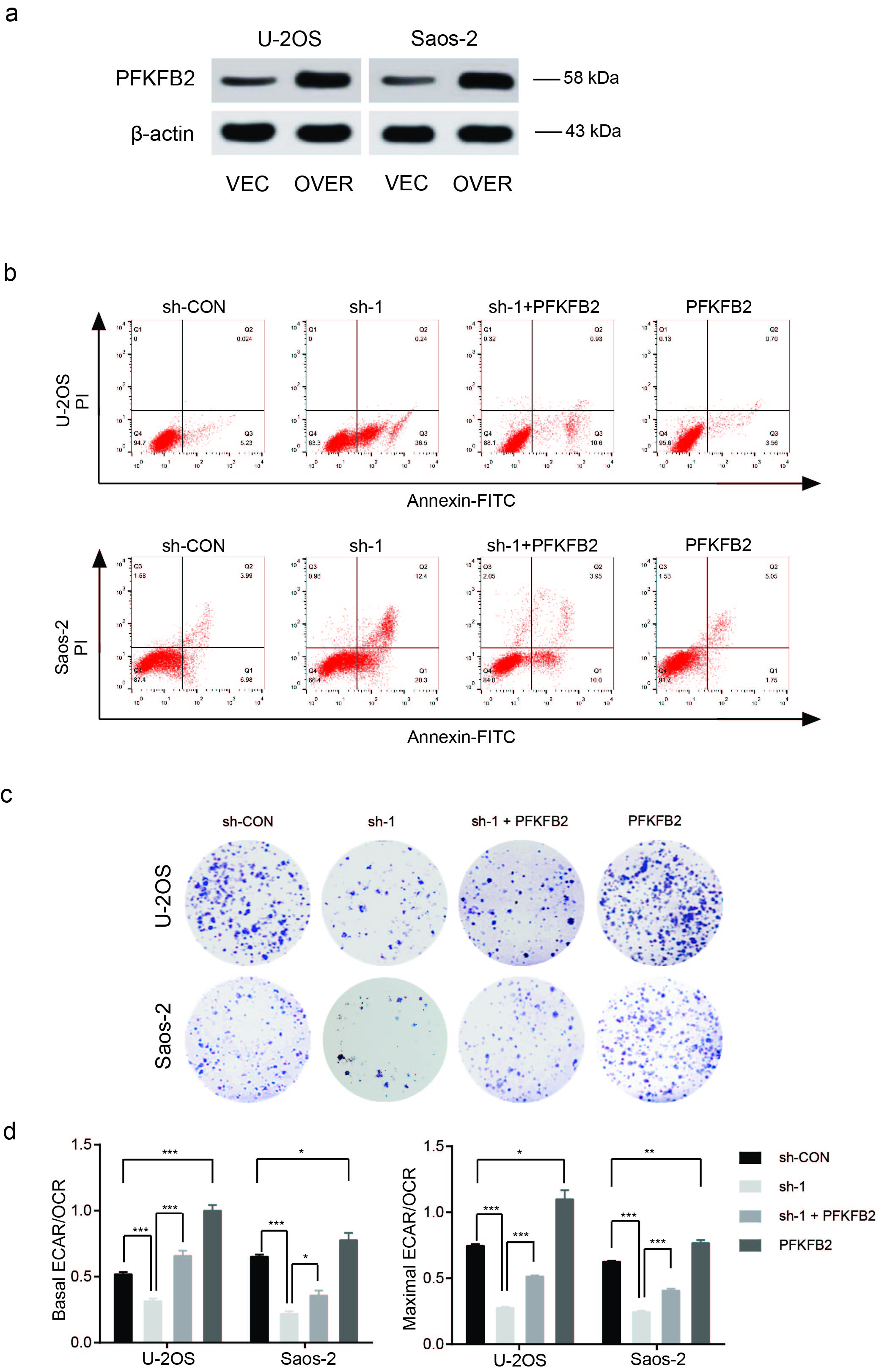

Supplement: Supplementary file 6 — Supplementary figure 5 [file 41419_2018_419_MOESM6_ESM.jpg]
